# Atomic Identification of Interfaces in Individual Core@shell Quantum Dots

**DOI:** 10.1002/advs.202102784

**Published:** 2021-10-13

**Authors:** Guiju Liu, Wenshuang Liang, Xuyan Xue, Federico Rosei, Yiqian Wang

**Affiliations:** ^1^ College of Physics & State Key Laboratory Qingdao University No. 308 Ningxia Road Qingdao 266071 P. R. China; ^2^ Centre Énergie Matériaux et Télécommunications Institut National de la Recherche Scientifique 1650 Boulevard Lionel‐Boulet Varennes Québec J3X 1S2 Canada

**Keywords:** CdSe@CdS core@shell quantum dots, crystal structure, interfaces, microstructures

## Abstract

CdSe@CdS Core@shell quantum dots (QDs) have been widely studied in recent years, due to their architecture which allows to tailor properties by controlling structure and composition. However, since CdSe and CdS have the same crystal structure, same cations, and similar lattice parameters, it is very challenging to image the interface. Herein, high‐resolution transmission electron microscopy, high‐angle annular dark‐field imaging, and energy‐dispersive X‐ray spectroscopy elemental mapping are combined to characterize the core@shell structure and identify the interface in the CdSe@CdS QDs with different CdS shell thicknesses. By examining changes in lattice spacing in an individual CdSe@CdS quantum dot, the atomic core@shell interface is identified. For thin‐shelled QDs, an ideal coherent interface forms between core and shell due to the small lattice mismatch, and the lattice spacing remains unchanged at the core and shell regions. For thick‐shelled QDs, the lattice spacing is different at the core and shell regions, while the heterostructured interface is still coherent and cannot be clearly imaged. As the shell thickness further increases, a sharp core@shell interface appears. The results define an approach to characterize the heterostructure of two materials with the same crystalline structure and cations.

## Graphical abstract

A simple method is reported to identify the interface in individual CdSe@CdS QDs. For thin‐shelled QDs, an ideal coherent interface forms between core and shell, and the lattice spacing remains unchanged. For thick‐shelled QDs, the lattice spacing is different at the core and shell regions. As the shell thickness further increases, a sharp core@shell interface appears.

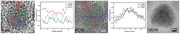

## 1 Introduction

Colloidal semiconductor quantum dots (QDs) have been widely studied for applications in solar cells, luminescent solar concentrators, photocatalysis, and other optoelectronic devices due to their high quantum yield, size/chemical composition tunable absorption and emission spectra, and solution processability.^[^ ^1^, ^2^, ^3^, ^4^ ^]^ As the most studied QDs, those with CdX (X = Se, S, Te) composition may crystallize in two different structures, that is, the cubic zincblende (ZB) and the hexagonal wurtzite (WZ) structure.^[^ ^5^, ^6^ ^]^ In these two structures, each cation is coordinated with four counter‐ions in the tetrahedral configuration and the local coordination and bond length are identical, while the stacking sequence of the two structures is different.^[^ ^6^, ^7^ ^]^ In the ZB structure, the lattice plane follows an ABCABC stacking along the [111] direction, while in the WZ structure, it follows an ABABAB stacking along the [0001] direction. This structural difference is subtle, thus it is difficult to synthesize nanocrystals with perfect crystallinity.^[^ ^7^, ^8^, ^9^, ^10^ ^]^ In addition, for traditional bare QDs, surface trap states/defects are unavoidable due to the small size and large specific surface area, which influence their optical properties. Coating the core QDs with a shell layer is a typical approach to passivate surface defects, which can reduce the surface effects on the optical properties of the QDs.^[^ ^11^, ^12^, ^13^, ^14^, ^15^ ^]^ Compared with bare QDs, core@shell QDs exhibit an enhanced photoluminescence quantum yield, higher efficiency, and stability within solar cells and other optoelectronic devices.^[^ ^16^, ^17^ ^]^ Among various core@shell QDs, CdSe@CdS is one of the most studied and best developed core@shell systems.

For CdSe@CdS core@shell QDs, CdSe core and CdS shell have the same crystalline structure and a small lattice mismatch (3.9%).^[^ ^18^ ^]^ Therefore, the CdS shell can grow epitaxially along the CdSe core. CdSe@CdS QDs have been successfully synthesized with control of size and shape by using several synthetic approaches, revealing unique structure/property relationships.^[^ ^19^, ^20^, ^21^ ^]^ For example, Chen et al.^[^ ^22^ ^]^ reported high‐quality CdSe@CdS QDs with narrow emission linewidths and suppressed blinking by using octanethiol and cadmium oleate as precursors for the growth of the CdS shell. Ghosh et al.^[^ ^23^ ^]^ reported the detailed growth of CdSe@CdS QDs and emphasized the effect of reaction parameters on their shape and crystalline phase, and explored the relationship between the structure and optical properties. However, due to the good lattice match between CdSe and CdS and the same cation of the two materials, it is very challenging to identify the interface between the core and shell by using traditional transmission electron microscopy (TEM). Imaging the interface in core@shell CdSe@CdS QDs is a long‐standing unresolved challenge, which limits in‐depth research on the relationship between the band alignment and properties, and their effective exploitation in optoelectronic devices.

Identifying the interface of CdSe@CdS core@shell QDs would help to determine the shell thickness, the shape, and position of the core, which play an important role in the optical properties of the QDs. Although descriptions of the CdSe@CdS core@shell structure have been reported in the literature, most of the information was derived from the size change of the nanocrystals compared with core QDs and the synthetic process.^[^ ^24^, ^25^ ^]^ For example, Li et al.^[^ ^20^ ^]^ synthesized high‐quality CdSe@CdS core@shell QDs using the successive ionic layer adsorption and reaction (SILAR) approach and achieved precise control of the shell thickness. Up to now, the interface between CdSe core and CdS shell in the CdSe@CdS QDs has not been identified at the atomic level. However, for QDs that have different cations in the core and shell, the exact atomic interface has been observed using high‐resolution TEM (HRTEM), such as PbSe@CdSe QDs,^[^ ^26^, ^27^ ^]^ PbTe@CdTe QDs,^[^ ^28^ ^]^ and PbS@CdS QDs.^[^ ^29^ ^]^ For CdSe@CdS core@shell QDs, only Tan et al.^[^ ^30^ ^]^ have observed the core@shell structure in hexagonal pyramid and hexagonal bipyramid CdSe@CdS QDs by energy‐dispersive X‐ray spectroscopy (EDS) elemental mapping in scanning TEM (STEM) mode. However, due to the challenges of microscopic imaging, the subtle structural parameters of the core@shell QDs system remain unquantified. The atomic‐scale characterization of the interface in an individual CdSe@CdS QD is still a formidable challenge. In particular, the exact atomic interface in an individual CdSe@CdS QD, which affects the band alignment and optical properties, is still poorly understood.

Here we synthesized CdSe@CdS core@shell QDs by a hot‐injection and SILAR approach. X‐ray diffraction (XRD), HRTEM, high‐angle annular dark‐field (HAADF), and EDS elemental mapping were used to analyze the crystallinity, microstructure, and core@shell structure of the QDs. Considering the different lattice parameters of CdSe and CdS, we propose a method to determine the core@shell interface of an individual CdSe@CdS QD in HRTEM and HAADF images, by measuring the variation of lattice spacing in different regions in a single nanocrystal. Combined with EDS elemental mapping, we are able to pinpoint the location of the CdSe core. The results provide a method to identify the core@shell structure or interface of heterostructured nanocrystals with the same cations and crystalline structure.

## 2 Results and Discussion

### 2.1 Microstructure of CdSe QDs

Extensive HRTEM observations of CdSe core QDs show that most nanocrystals (≈90%) exhibit a perfect hexagonal WZ structure, while some planar defects such as stacking faults are also observed (Figure S1, Supporting Information). In addition to the internal defects, because of the small size and large specific surface area of the QDs, surface atoms without ligand protection will exist as surface defect states, which can easily capture excitons thereby affecting the luminescent properties and stability.^[^ ^31^ ^]^ To reduce the influence of surface defect states on the properties, a CdS shell layer was coated on the CdSe core. The band gap values of bulk CdSe and CdS are 1.74 and 2.49 eV, respectively.^[^ ^32^, ^33^ ^]^ When the CdSe surface is coated with a CdS shell, an energy barrier can form between the excitons and surface defects, which decreases the probability of surface defects capturing electrons or holes, reducing the sensitivity of QDs to the surface environment and enhancing the optical properties.

### 2.2 Crystal Structure and Morphology of CdSe and CdSe@CdS QDs

**Figure 1:**
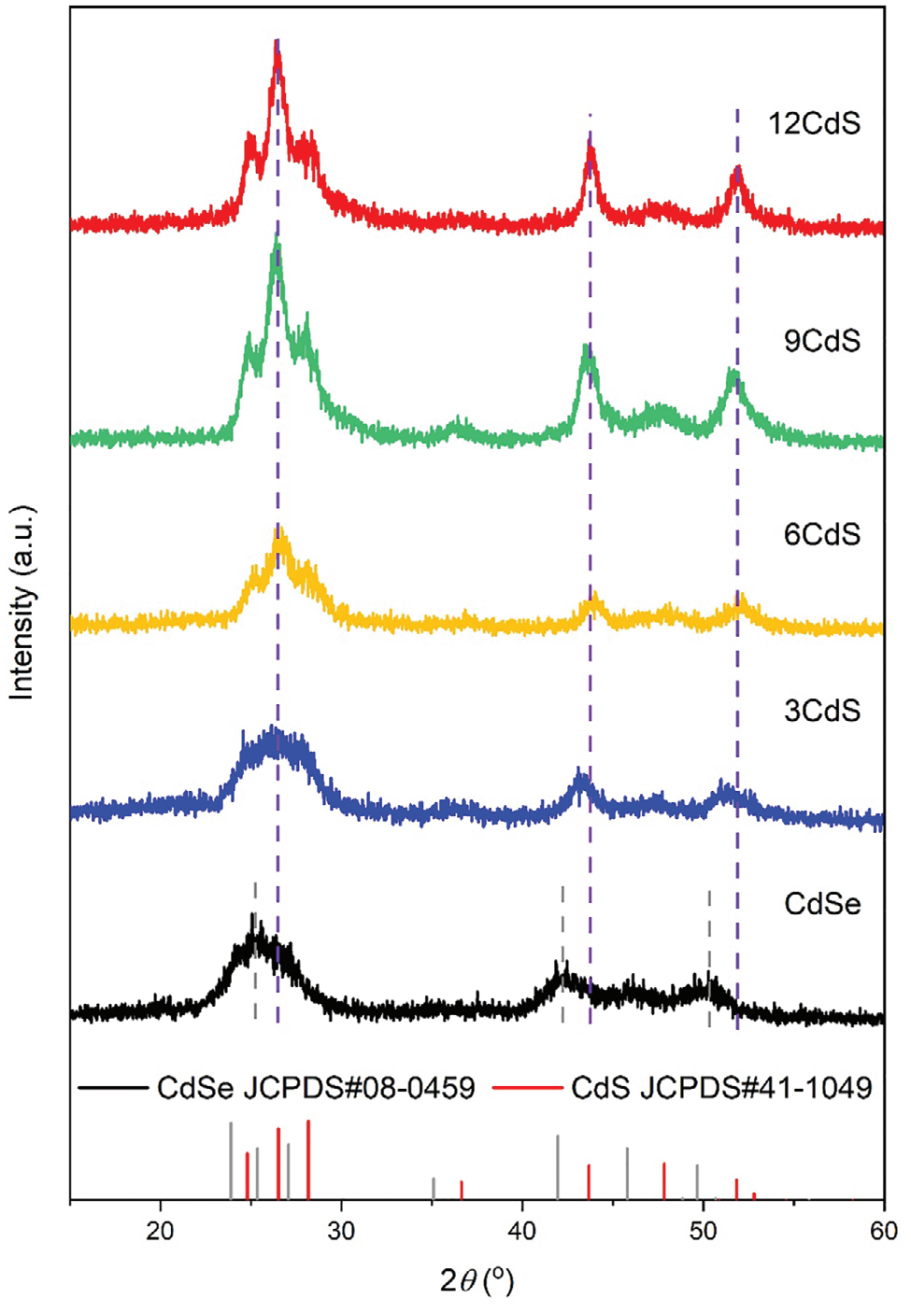
XRD patterns of bare CdSe, CdSe@3CdS, CdSe@6CdS, CdSe@9CdS, and CdSe@12CdS QDs, respectively.

XRD patterns were acquired to elucidate the crystalline phases of the synthesized QDs with different shell thicknesses. **Figure** **1** displays XRD patterns of CdSe@CdS QDs with different CdS shell thicknesses (0, 3, 6, 9, and 12 monolayers). A peak at ≈45.8^o^ is visible in the XRD pattern of CdSe QDs, which corresponds to the (10$\bar{1}$3) plane of hexagonal WZ CdSe. As the cubic ZB structure has no diffraction peak at this position, it is concluded that the bare CdSe QDs have a hexagonal WZ structure. The CdSe@CdS QDs with different shell thicknesses (3, 6, 9, and 12 monolayers) also show a hexagonal WZ structure. The vertical dashed lines in Figure 1 show that the coating of the CdS shell shifts the diffraction peaks towards higher angles, compared with the bare CdSe QDs. In addition, when the shell layer reaches a certain thickness (six monolayers of CdS), the diffraction peaks no longer shift, as shown by the vertical purple dashed lines. Meanwhile, when increasing the shell thickness, the size of the QDs increases, the crystallinity of the QDs improves and the separation of the first three peaks is more prominent, consistent with previous reports.^[^ ^34^, ^35^ ^]^ For the CdSe@CdS QDs with thick shell, there is no obvious structural information of the CdSe core due to its small volume. For instance, for the CdSe core QDs with a diameter of 4 nm, when coated with 6 monolayers of CdS shell, the shell volume accounts for more than 87% of the whole QDs. Therefore, the shell materials occupy the main part of the QDs, the overall lattice parameters are close to those of the CdS shell, and the crystal structure and lattice parameters no longer change.

**Figure 2:**
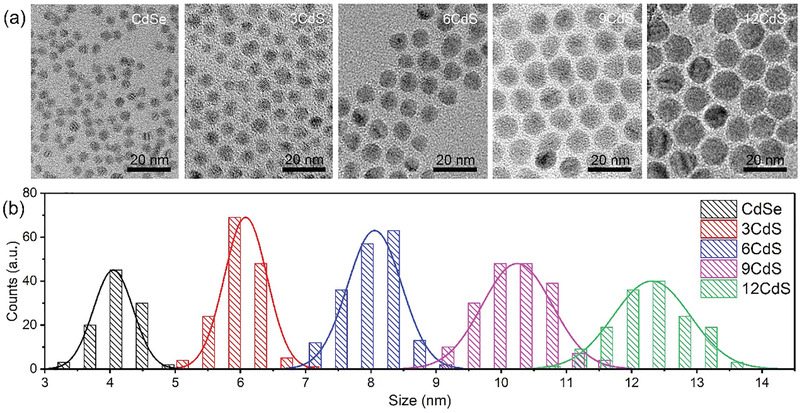
Morphology and size distribution of the CdSe@CdS QDs. a) Bright‐field TEM images and b) size distribution diagrams of the CdSe@CdS QDs with different CdS shell thicknesses.

**Figure** **2**a shows typical bright‐field TEM images of CdSe@CdS QDs with different CdS shell thicknesses (0, 3, 6, 9, and 12 monolayers). All these QDs present monodispersibility and uniform morphology. When increasing the CdS shell thickness, the QDs’ shape transforms from nearly spherical to a faceted morphology. When increasing the number of CdS monolayers, the size of the as‐synthesized QDs increases almost linearly, as shown in Figure 2b. The average diameter of bare CdSe QDs is 4.05 nm, and after coating 3, 6, 9, and 12 monolayers of CdS shell, the average diameter increases to 6.08, 8.06, 10.25, and 12.31 nm, respectively, consistent with theoretical values calculated using the known core size and assumed shell thickness (1 monolayer = 0.3375 nm^[^ ^23^ ^]^).

### 2.3 Microstructure of CdSe@CdS QDs

**Figure 3:**
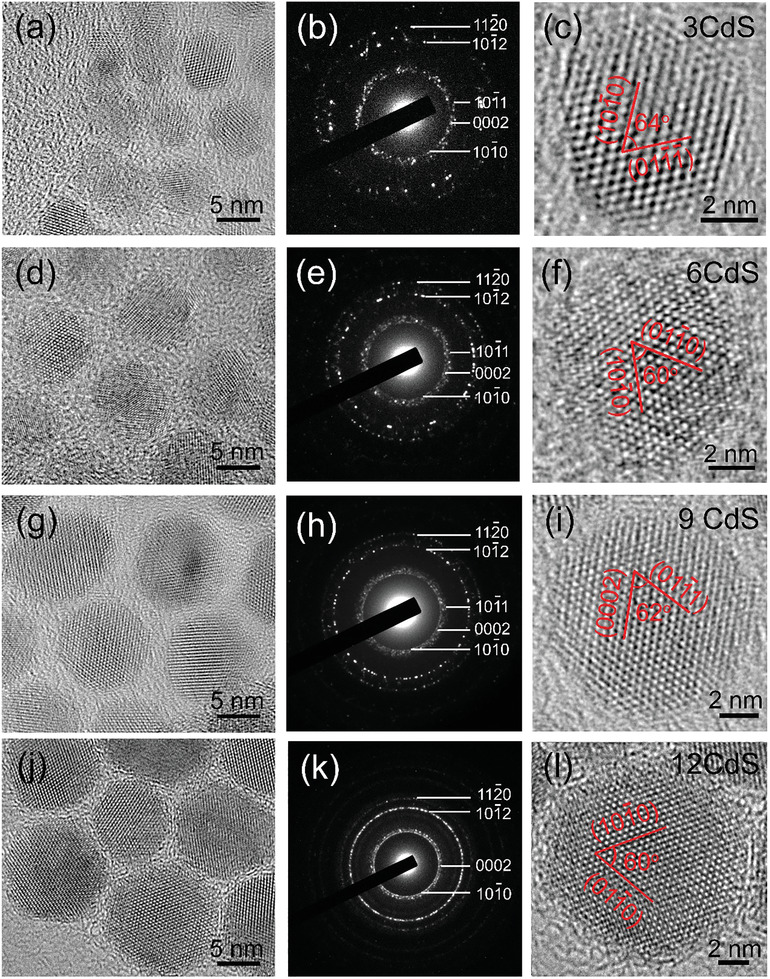
HRTEM images and SAED patterns of CdSe@CdS QDs. a–c) CdSe@3CdS QDs, d–f) CdSe@6CdS QDs, g–i) CdSe@9CdS QDs, and j–l) CdSe@12CdS QDs.

Extensive TEM imaging was carried out to investigate the microstructure of these CdSe@CdS QDs in detail. **Figure** **3** shows typical HRTEM images and selected‐area electron diffraction (SAED) patterns of CdSe@CdS QDs after coating with 3, 6, 9, and 12 CdS monolayers. The HRTEM images (Figures 3a,d,g,j) show that all the core@shell QDs exhibit clear lattice fringes that run through the entire particle, indicating that the CdS shell grows epitaxially on the CdSe core.^[^ ^36^ ^]^ In Figure 3b, the three adjacent diffraction rings in the innermost layer of the SAED pattern confirm that the CdSe@3CdS QDs have a hexagonal WZ structure. Figure 3c shows a typical HRTEM image of an individual CdSe@3CdS QD. Two planes with an angle of 64^o^ and lattice spacings of 3.70 and 3.35 Å correspond to the (10$\bar{1}$0) and (01$\bar{1}\bar{1}$) planes of hexagonal WZ CdSe. Similar to Figure 3b, the SAED pattern in Figure 3e confirms the WZ structure for the CdSe@6CdS QDs. In the case of individual CdSe@6CdS QD in Figure 3f, the measured lattice spacings of 3.58 Å and 3.58 Å with an angle of 60^o^ between the two planes are consistent with the (10$\bar{1}$0) and (01$\bar{1}$0) planes of WZ CdS. The SAED pattern in Figure 3h indicates that the CdSe@9CdS QDs still maintains the WZ structure. Figure 3i shows that the two planes with an angle of 62^o^ and lattice spacings of 3.28 and 3.40 Å are consistent with the (01$\bar{1}$1) and (0002) planes of WZ CdS. Figure 3l shows a typical HRTEM image of an individual CdSe@12CdS QD viewed along [0001] direction. Similar to Figure 3f, the two planes with an angle of 60° correspond to the (10$\bar{1}$0) and (01$\bar{1}$0) planes of WZ CdS. All the SAED patterns of the QDs (Figures 3b,e,h,k) exhibit hexagonal diffraction rings. When increasing the shell thickness, the diffraction rings become more pronounced, indicating that the synthesized CdSe@CdS QDs mainly have a hexagonal structure, which is consistent with the XRD patterns (Figure 1). Both CdSe core and CdS shell have a hexagonal WZ structure, therefore, the interface between the core and shell cannot be clearly observed in the HRTEM images of CdSe@CdS QDs.

**Figure 4:**
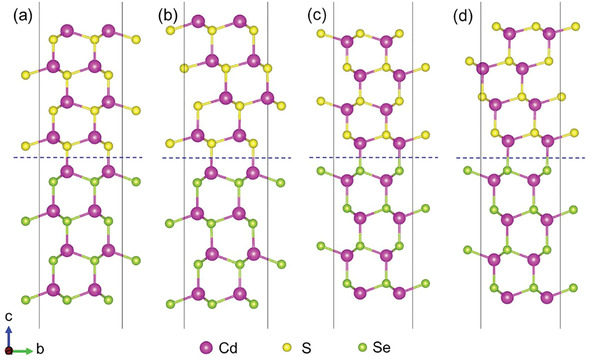
The interface model for CdSe/CdS with different crystal structures. The interface model for CdSe/CdS with crystal structures of a,c) WZ/WZ and b,d) WZ/ZB. The exposed surfaces of CdSe are terminated by Cd atoms in (a,b) and Se atoms in (c,d).

To understand the epitaxial growth of the CdS shell on CdSe core, we investigated the interfacial stability between the WZ CdSe and WZ (or ZB) CdS interfaces by calculating the formation energies of WZ (or ZB) CdS on WZ CdSe using cambridge sequential total energy package (CASTEP) software. Considering that CdS can exist in two crystal structures (WZ and ZB), the structure of the CdS shell on the WZ CdSe core may be different. We constructed four slab interface models of WZ CdSe (0001) planes and CdS, as shown in **Figure** **4**. The bottom slabs are CdSe and the upper slabs are CdS. Figures 4a,b are the interface models for WZ CdSe with Cd atoms as termination layer coupled with S atoms in WZ CdS and ZB CdS, respectively. Figures 4c,d display the interface models for WZ CdSe with Se atoms as termination layer coupled with Cd atoms in WZ CdS and ZB CdS, respectively. After structural optimization, the lattice mismatch between WZ CdSe and WZ CdS is 2.98%, while for the WZ CdSe and ZB CdS, the lattice mismatch is 3.37%. The formation energies for the four interface models were estimated using first‐principles density functional theory calculations.^[^ ^37^ ^]^ In the calculations, the ultrasoft pseudopotential plane‐wave was used to describe the interaction between valence electrons and ionic solids, and the exchange‐correlation potential is treated by the Perdew–Burke–Ernzerhof functional within the generalized gradient approximation. To prevent the influence of periodic adjacent interfaces, a vacuum layer with a thickness of 10 Å was added to the interface and surface models. The interface binding energy is an important factor to measure the binding ability of two phases, which can indicate the stability of the interface structure. It can be calculated using the following equation:^[^ ^38^ ^]^

(1) $$E_{\mathrm{coh}}=\;\left(E_{\mathrm{CdSe}/\mathrm{CdS}}^{\mathrm{total}}-E_{\mathrm{CdSe}}^{\mathrm{slab}}-E_{\mathrm{CdS}}^{\mathrm{slab}}\right)/A$$

where $E_{\mathrm{CdSe}/\mathrm{CdS}}^{\mathrm{total}}$ is the total energy of the CdSe@CdS interface, $E_{\mathrm{CdSe}}^{\mathrm{slab}}$ is the energy of the surface configuration of WZ CdSe (0001) plane, $E_{\mathrm{CdS}}^{\mathrm{slab}}$ is the energy of the surface configuration of the WZ CdS (0001) plane or the ZB CdS (111) plane. Through the above equation, the interface binding energies of the four interface models are −1.656, −1.589,−1.368, and −1.2000 J m^−2^, respectively. Irrespective of whether the CdSe is terminated with Cd or Se atoms, the interface formed by the WZ CdSe and WZ CdS is more stable, indicating that it is easier to form the structure of WZ CdS in the process of coating CdS on WZ CdSe. This can account for the epitaxial growth of the WZ CdS on the WZ CdSe with no distinct interface in our HRTEM observations.

To verify the core@shell structure of the CdSe@CdS QDs, we used EDS elemental mapping in STEM mode to obtain the atomic distributions of Cd, Se, and S elements. Figures S2 and S3, Supporting Information, show the HAADF images and EDS mapping of CdSe@3CdS QDs and CdSe@9CdS QDs. The Cd and S elements are distributed throughout the whole QDs while the Se element is only present in the core region of the QDs, which intuitively proves the core@shell structure of the synthesized CdSe@CdS QDs. However, due to the small size of the QDs and the resolution limit of the EDS mapping, the atomic interface between CdSe and CdS remains unclear.

**Figure 5:**
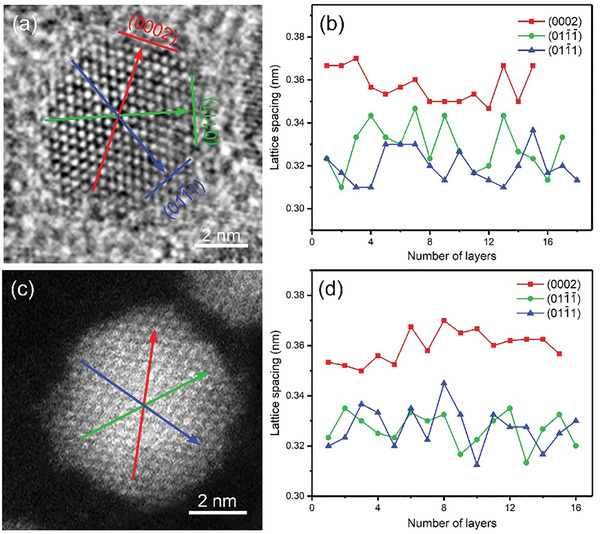
Microstructure of an individual CdSe@3CdS QD. a) HRTEM image and b) lattice spacing variation, c) HAADF image, and d) lattice spacing change of an individual CdSe@3CdS QD.

To identify the core@shell interface of the CdSe@CdS QDs, we analyzed the detailed structure of an individual QD. **Figure** **5**a shows a typical HRTEM image of an individual CdSe@3CdS QD. The measured lattice spacing of 3.57 Å corresponds to the (0002) planes of WZ CdSe, and the other two planes with average lattice spacings of 3.27 and 3.18 Å correspond to the (01$\bar{1}$1) and (01$\bar{1}\bar{1}$) planes of WZ CdSe, respectively. Figure 5b shows the change of lattice spacing from one edge to the opposite edge in the nanoparticle along the direction of the three planes, indicated by red, green, and blue arrows, respectively. There are no obvious changes in the lattice spacing at the core and edge regions of the QDs. In the initial stages of shell growth, the lattice strain is accommodated by elastic deformation. Thus, no difference in the planar spacing is observed for the QDs with thin shell layer. HAADF images were acquired to observe the difference in QDs (Figure 5c). Similar to Figure 5a, the variation of lattice spacing was also measured from one edge to the opposite edge along the three directions in Figure 5c. As shown in Figure 5d, no obvious changes can be found in the lattice spacing at core and edge regions, which is consistent with the HRTEM results (Figure 5b). However, for the HAADF image, the intensity of every atomic column is approximately proportional to Z^1.67^ (Z is the atomic number), thus, it can provide key information regarding the elemental distribution within nanostructures at sub‐Å resolution.^[^ ^39^ ^]^ In Figure 5c, the contrast of the core is brighter due to the heavier atomic weight of Se, whereas the shell is darker due to the lower atomic weight of S, confirming the core@shell structure of CdSe@3CdS QD. However, the interface of the core@shell structure is still indistinct. To distinguish the core@shell structure more clearly, we further examined the CdSe@CdS QDs with thicker shells.

**Figure 6:**
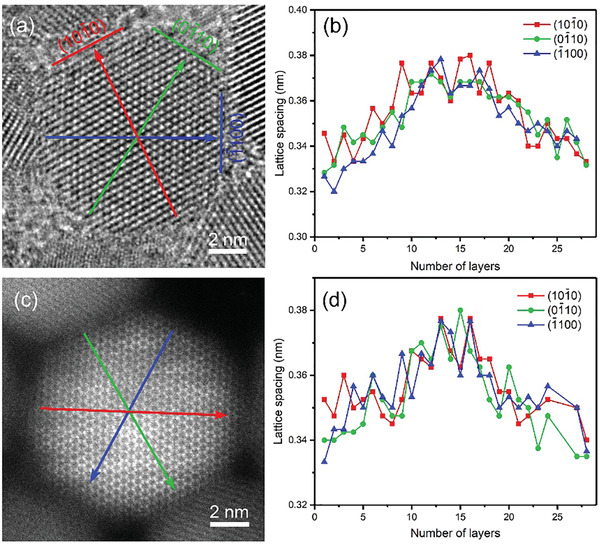
Microstructure of an individual CdSe@9CdS QD. a) HRTEM image and b) lattice spacing variation, c) HAADF image, and d) lattice spacing change of an individual CdSe@9CdS QD.

**Figure** **6**a shows the HRTEM image of an individual CdSe@9CdS QD. The measured average lattice spacing of 3.54 Å corresponds to the (10$\bar{1}$0) plane of WZ CdSe@CdS, and the other two planes with an angle of 60^o^ with (10$\bar{1}$0) plane correspond to (0$\bar{1}$10) and ($\bar{1}$100) planes of WZ CdSe@CdS, respectively. Figure 6b shows the variation of lattice spacing from one edge to center and to opposite edge along three different directions in Figure 6a, indicated by red, green, and blue arrows, respectively. The lattice spacings of the three planes all show a trend of first increase and then decrease from the 1st layer at one edge to the center and then to the 27th layer at the opposite edge, which can be used to infer the interface position of the CdSe@9CdS QD. For the CdSe@3CdS QDs, the lattice spacing for a certain plane remains nearly constant at the core and shell regions. In the case of CdSe@9CdS QD, the lattice spacing in the central region is larger than that at the edges. This is due to the fact that in the early stages of CdS shell coating, the shell grows epitaxially along the hexagonal structure of CdSe. The elastic deformation accommodates the lattice strain, thus, the crystal lattice spacing of the core is maintained and the interface between the core and shell is an ideal coherent interface. As the shell thickness increases, the lattice strain increases and the shell tends to grow with its intrinsic lattice parameters. The lattice parameters of CdSe are larger than those of CdS, therefore, from the center to edge regions, the lattice spacing decreases, further confirming the core@shell structure of the CdSe@CdS QDs. Figure 6c shows a typical HAADF image of an individual CdSe@9CdS QD. In this image, it can be clearly seen that each hexagon is composed of six adjacent atomic columns. The contrast of the core region is brighter than that of the edges, which may be attributed to the difference in atomic number of Se and S, further confirming the core@shell structure of the CdSe@9CdS QDs. The variation of the lattice spacing can be obtained by measuring the lattice spacing of every atomic layer from one edge to the opposite edge along the three directions in Figure 6c, as shown in Figure 6d. Consistent with Figure 6b, the lattice spacing exhibits obvious changes in the core and shell regions, which allows to deduce the core@shell interface position of CdSe@9CdS QD. Although the lattice spacing varies in different regions for CdSe@9CdS QD, the shell layer still epitaxially grows along the core, and due to the small lattice mismatch between CdSe and CdS, the interface is still coherent and cannot be clearly identified.

**Figure 7:**
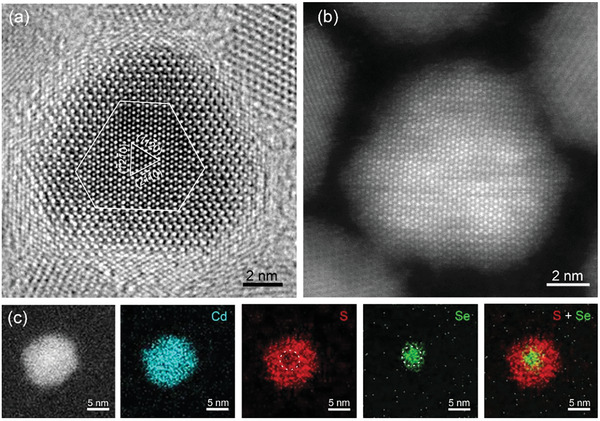
Microstructure of an individual CdSe@12CdS QD. a) HRTEM image, b) HAADF image, and c) EDS elemental mapping of an individual CdSe@12CdS QD.

By further increasing the shell thickness, we analyzed the microstructure of an individual CdSe@12CdS QD using HRTEM, HAADF, and EDS elemental mapping, as shown in **Figure** **7**. The HRTEM image (Figure 7a) shows an obvious contrast difference between the core and shell regions, which proves the core@shell structure of CdSe@12CdS QD. The interface between the core and shell is indicated by white lines. In Figure 7a, the QD is viewed along the [0001] direction, and the three labeled planes with an angle of 60^o^ to each other are consistent with the {11$\bar{2}$0} planes of WZ CdSe. Figure 7b shows the HAADF image of the CdSe@12CdS QD. Since the intensity of every atomic column is proportional to Z^1.67^, the brighter contrast at the core region can be attributed to the higher atomic number of Se as opposed to S. To prove the core@shell structure intuitively, we acquired EDS maps of individual QDs to compare the elemental distribution of Cd, S, and Se, as shown in Figure 7c. The Cd and S atoms are distributed throughout the whole QD, while atomic Se is located in the center, similar to Figure S3, Supporting Information. In addition, the contrast at the center region of the QDs is darker than that at the edges (labeled by a white circle), indicating that there is less atomic S at the core region. By overlapping the S and Se dispersion, we observe that Se is distributed in the core region while S is mainly distributed around the Se, demonstrating the core@shell structure of CdSe@12CdS QD. Compared with the HRTEM and HAADF images of CdSe@3CdS, CdSe@9CdS, and CdSe@12CdS QDs (Figures 5, 6, 7), we conclude that the interface of the CdSe@CdS core@shell QDs can be observed under certain conditions (thick‐shell QDs and [0001] zone‐axis). The shell thickness of the QDs affects the observation of core@shell structures. When the CdS shell thickness increases, the lattice spacing of the CdS shell tends to approach that of bulk CdS, and the difference in lattice spacing between the core and shell increases. As the shell thickness further increases, the difference in lattice spacing may disappear due to the very small volume of the core.

### 2.4 Discussion

**Figure 8:**
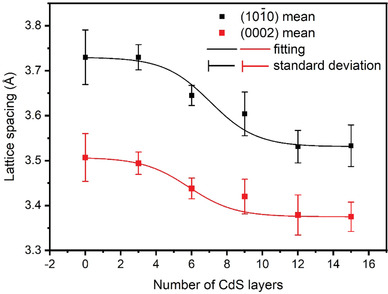
The change trend of average spacing for (10$\bar{1}$0) and (0002) planes in CdSe@CdS QDs with different shell thicknesses. The square dots represent the average lattice spacing, and the error bars are the standard deviations for each set of measurements.

By measuring the lattice spacing of various CdSe@CdS QDs, we compared the change in average lattice spacing for (0002) and (10$\bar{1}$0) planes with different shell thicknesses, as shown in **Figure** **8**. At the initial stage of CdS shell growth, the lattice spacings for both (0002) and (10$\bar{1}$0) planes are very similar to that of CdSe. When increasing the CdS shell thickness, the lattice spacing decreases due to the smaller lattice parameter of CdS compared to CdSe. When the CdS shell grows thicker, the lattice spacing is closer to that of bulk CdS. Based on the calculation of critical thickness, this phenomenon may be attributed to the change of strain release mode. For the initial growth of the CdS shell (<5 monolayers), since the strain caused by the lattice mismatch is accommodated by elastic deformation, CdS grows epitaxially along the lattice of CdSe. Thus, a coherent interface forms between CdSe and CdS, and the lattice spacing is close to that of CdSe. When increasing the shell thickness, the strain cannot be relieved by the elastic deformation, thus the lattice spacing difference between core and shell will increase and the intrinsic lattice parameter of CdS will gradually become dominant. When the shell layer exceeds a certain thickness (9 monolayers), the lattice spacing of the QDs approaches the lattice parameter of bulk CdS due to the very small volume of the core compared to the shell.

From the above analysis, we infer that for the CdSe@CdS QDs with a thin shell thickness, the interface between core and shell is coherent with no change in lattice spacing, while when the CdS shell layer increases to a certain thickness, although the interface is still coherent, the lattice spacing of the shell will be different from the one of the core region.

In previous reports on heterointerfaces, the interface is usually accompanied by the periodic arrangement of misfit dislocations, leading to the formation of a semi‐coherent interface. However, the interface is coherent between two misfit dislocations.^[^ ^40^ ^]^ The misfit dislocation spacing (*d*_s_) is determined by the lattice mismatch (*f*) and lattice spacing (*d*), which can be expressed as: *d_s_* = *d*/*f*.^[^ ^40^ ^]^ In the case of CdSe@CdS core@shell spherical nanoparticles, their projection along any direction is circular (Figure S4, Supporting Information). When the circumference difference of shell and core exceeds the misfit dislocation spacing, the ideal coherent interface will be destroyed. Thus, the critical thickness of the shell, *h*_c_, where the lattice spacing difference begins to appear, can be obtained using the following formula:

(2) $$h_{c}=\frac{d_{CdS}}{2\pi f}\;$$

where *d*_CdS_ is the lattice spacing of the CdS shell, and *f* is the lattice mismatch between the core and shell along a certain direction (see Supporting Information for details). For the (10$\bar{1}$0) plane, *d*_CdS_ is 3.58 Å and *f* is 3.76%. The *h*_c_ for CdSe@CdS is calculated to be 1.52 nm, corresponding to 5 monolayers of CdS. Thus, for the CdSe@CdS QDs, the strain caused by the lattice mismatch is accommodated by elastic deformation when the CdS shell thickness is less than 5 monolayers. In addition, we found that when the shell thickness is between *h*_c_ and 2*h*_c_, the core and shell show different lattice spacing. When further increasing the shell thickness (>2*h*_c_), a sharp interface between the core and shell will appear.

## 3 Conclusions

In summary, we synthesized CdSe@CdS core@shell QDs with different shell thicknesses by hot‐injection and SILAR approaches. XRD and HRTEM results indicate that the CdSe QDs and CdSe@CdS QDs all crystallize in the WZ structure. Theoretical calculations confirm that the interface for the WZ CdSe/WZ CdS structure is more stable than the one of the WZ CdSe/ZB CdS structure. HRTEM, HAADF, and EDS elemental mapping were used to directly demonstrate the formation of the core@shell structure for CdSe@CdS QDs. The CdSe core and CdS shell have the same crystal orientation with a seamless match of their crystal lattices. Due to the different lattice parameters of CdSe and CdS, lattice spacing measurements in HRTEM and HAADF images of one nanoparticle were used to demonstrate the formation of the CdSe@CdS core@shell structure and to identify the core@shell interface of the QDs. For the thin‐shelled QDs (<5 monolayers), an ideal coherent interface was observed between the core and shell, and no lattice spacing variation was found. In the case of thick‐shelled QDs, the lattice spacing in the core is larger than that of the shell, while the heterostructured interface is still coherent. The core@shell interface of the thick‐shelled QDs can be determined by comparison with the change of lattice spacing. When further increasing the shell thickness (>9 monolayers), a sharp interface may be found between the core and shell. Our work defines a method to identify the interface of the core@shell structure or other heterostructure nanoparticles with the same cations and crystal structure. This method can be broadly extended to the characterization of other core@shell QDs, for example, PbSe@PbS, CdTe@CdSe, and CdSeTe@CdS QDs, and can be widely used by a broad community of scientists working in materials science, physics, and chemistry.

## 4 Experimental Section

### Synthesis of CdSe QDs

CdSe QDs were synthesized using a hot injection approach.^[^ ^23^ ^]^ Typically, Cd‐oleate (0.38 mmol), Trioctyl phosphine oxide (TOPO, 1 g), and octadecene (ODE, 8 mL) were mixed in a 100 mL round‐bottom flask. The reaction system was degassed at 110 °C for 30 min, then the temperature was increased to 350 °C under nitrogen atmosphere. Subsequently, the mixture of trioctyl phosphine (TOP) ‐Se (4 mmol, 4 mL), oleylamine (OLA, 3 mL), and ODE (1 mL) was quickly injected into the reaction system. The reaction temperature was then decreased to 320 °C. After several minutes, the solution was quickly cooled down to room temperature, and the CdSe QDs were collected by centrifugation with ethanol.

### Synthesis of CdSe@CdS core@shell QDs

The synthesis of CdSe@CdS core@shell QDs was carried out using the SILAR method.^[^ ^20^ ^]^ Precursor solutions of 0.2 M Cd‐oleate in ODE and 0.2 M sulfur dissolved in ODE were used for shell growth. In a 100 mL round‐bottom flask, 5 mL of OLA, 5 mL of ODE, and 2 × 10^−7^ mol of CdSe cores were degassed at 110 °C for 30 min. The reaction flask was then re‐stored with N_2_ and the temperature was raised to 240 °C under stirring. Subsequently, 0.41 mL of Cd‐oleate precursor solution was added dropwise and allowed to react for 1 h, followed with dropwise addition of S precursor solution (0.41 mL) for 10 min to complete the growth of one CdS monolayer. The Cd‐oleate and S precursor solutions were then alternately injected for 1 h and 10 min, respectively, to form the CdSe@CdS core@shell QDs with a certain shell thickness (3, 6, 9, and 12 monolayers of CdS). The additional volume of Cd‐oleate/S precursors for growing each monolayer shell was calculated according to the volume increment of each monolayer shell. After shell growth, the reaction system was cooled down to room temperature and collected by centrifuge tubes. The QDs were then purified by centrifugation with ethanol and dispersed in toluene for further characterization.

### Materials Characterization

XRD patterns were acquired using a Rigaku Ultima IV XRD diffractometer using a Cu‐K_*α*_ radiation source (*λ* = 1.5406 Å). TEM, HRTEM, and SAED images were acquired using a JEOL JEM 2100Plus TEM with a voltage of 200 kV. HAADF images and EDS elemental maps of multiple QDs were acquired using a Talos F200X TEM with a voltage of 200 kV. EDS elemental maps of the individual QD were measured using a JEM‐F200 Multi‐purpose Electron Microscope with a voltage of 200 kV. The CASTEP software was used to simulate the binding energy of the CdSe/CdS interface with different crystalline structure.

### Statistical Analysis

The data of lattice spacings presented in Figure 8 were obtained by measuring more than 10 nanoparticles. The average lattice spacing and the standard deviation were calculated using Microsoft Excel. The formula for calculation of standard deviation is s = sqrt{[(*x*_1_−*x*)^2^ +…(x*_n_*−*x*)^2^]/*n*}, where *x* is the average value of the corresponding planes, and *x*_1_ to *x_n_* are the measured values.

## Conflict of Interest

The authors declare no conflict of interest.

## Author Contribution

G.J.L. synthesized the materials, measured the XRD patterns, and analyzed the data. W.S.L. carried out the TEM measurements. X.Y.X. performed the calculations and wrote the calculation results. G.J.L., Y.Q.W., and F.R. wrote the manuscript with contributions from all authors.

## Supporting information

Supporting Information

## Data Availability

Research data are not shared.
