# Supplementary material for: Atomic Identification of Interfaces in Individual Core@shell Quantum Dots
Source: Adv Sci (Weinh). 2021 Oct 13;8(22):2102784. doi: 10.1002/advs.202102784 (PMC8596122; doi:10.1002/advs.202102784)
Supplement: Supplementary file 1 — Supporting Information [file ADVS-8-2102784-s001.pdf]

© 2021 Wiley-VCH GmbH

ADVANCED SCIENCE
Open Access

Supporting Information

for *Adv. Sci.,* DOI: 10.1002/advs.202102784

Atomic Identification of Interfaces in Individual Core/shell Quantum Dots

Guiju Liu, Wenshuang Liang, Xuyan Xue, Federico Rosei*, Yiqian Wang**

Atomic Identification of Interfaces in Individual Core/shell Quantum Dots

Guiju Liu, Wenshuang Liang, Xuyan Xue*, Federico Rosei*, Yiqian Wang*

Dr. G.J. Liu, W. S. Liang, Prof. X. Y. Xue, Prof. Y. Q. Wang

College of Physics & State Key Laboratory, Qingdao University, No. 308 Ningxia Road, Qingdao 266071, P. R. China

Email: xuexy@qdu.edu.cn, yqwang@qdu.edu.cn

Prof. F. Rosei

Centre Énergie Matériaux et Télécommunications, Institut National de la Recherche Scientifique, 1650 Boulevard Lionel-Boulet, Varennes, Québec, J3X 1S2 Canada

Email: federico.rosei@inrs.ca

1. Figures

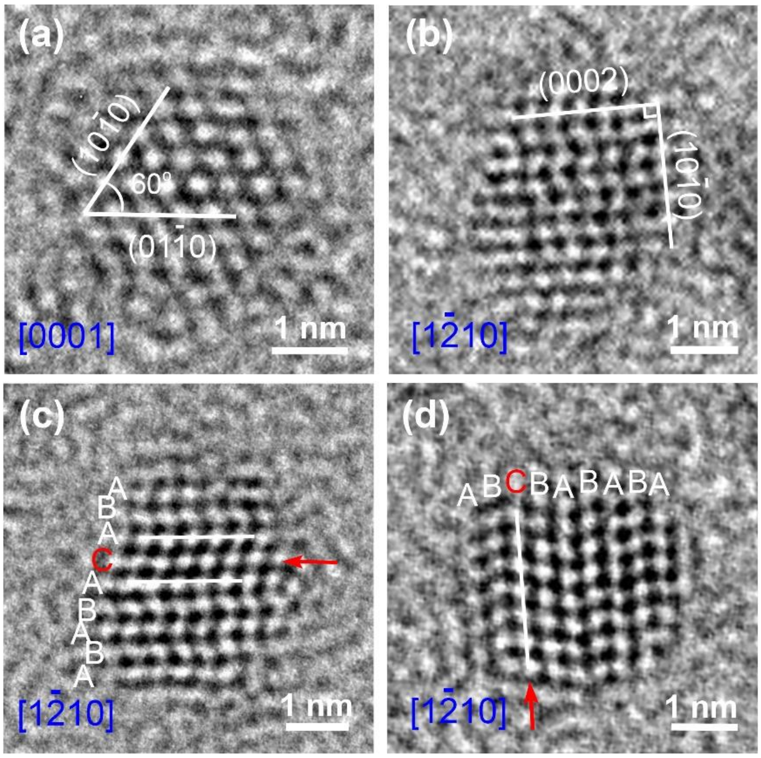

Figure S1. HRTEM images of an individual CdSe QDs. (a,b) Perfect WZ crystal structureof CdSe QDs under [0001] and [1$\bar{2}$10] zone-axis, respectively. (c,d) CdSe QDs with stacking faults.

Figure S1a,b are the typical HRTEM images of the synthesized CdSe core QDs with a perfect WZ structure. In Figure S1a, the particle was viewed along the [0001] zone axis. The two planes with an angle of 60° correspond to the (10$\bar{1}$0) and (01$\bar{1}$0) planes of the WZ structure of CdSe. In Figure S1b, the measured lattice spacing of two perpendicular planes is 3.72 Å and 3.56 Å, corresponding to the (10$\bar{1}$0) and (0002) planes of WZ CdSe, suggesting that this nanocrystal is viewed along the [1$\bar{2}$10] zone axis. Figures S1c,d are the small amount of CdSe QDs with stacking faults. Figure S1c exhibits a extrinsic stacking fault in an individual CdSe nanoparticle. A layer of atoms was inserted into the perfect-crystal layer sequence of …ABABAB…to form

an …ABABA**C**ABAB… structure. This stacking fault makes the lattice structure of a local cubic ZB structure appear in the hexagonal WZ structure. Similarly, in Figure S1d, the nanoparticle shows the extrinsic stacking faults that transforms the layer sequence into…ABABAB**C**BA….., where local twinning structure with only two atomic planes is formed. The presence of these inner defects is due to the fact that during the QDs synthesis, it is difficult to achieve a complete homogeneity reaction system. The ligand concentration, reaction temperature and other conditions will affect the crystalline structure of the synthetic product. Under the influence of stress, the energy in some regions in the reaction system is higher, which usually forms defects to release stress.

Figure S2 displays the HAADF image and EDS elemental mapping of CdSe@3CdS QDs, allowing to identify the distribution of Cd, S and Se elements. Figures S2e,f report the elemental maps after superimposing the distribution of Cd and S elements, and Cd, S and Se elements, respectively. From the overlapped maps, we deduce that the Cd and S elements are distributed in the whole QDs while Se is present in the core region of the QDs, demonstrating the core/shell structure of the synthesized CdSe/3CdS QDs. However, due to the small size of the QDs and the low amount of Se and S atoms, the interface between QDs and the core/shell interface is not clearly visible.

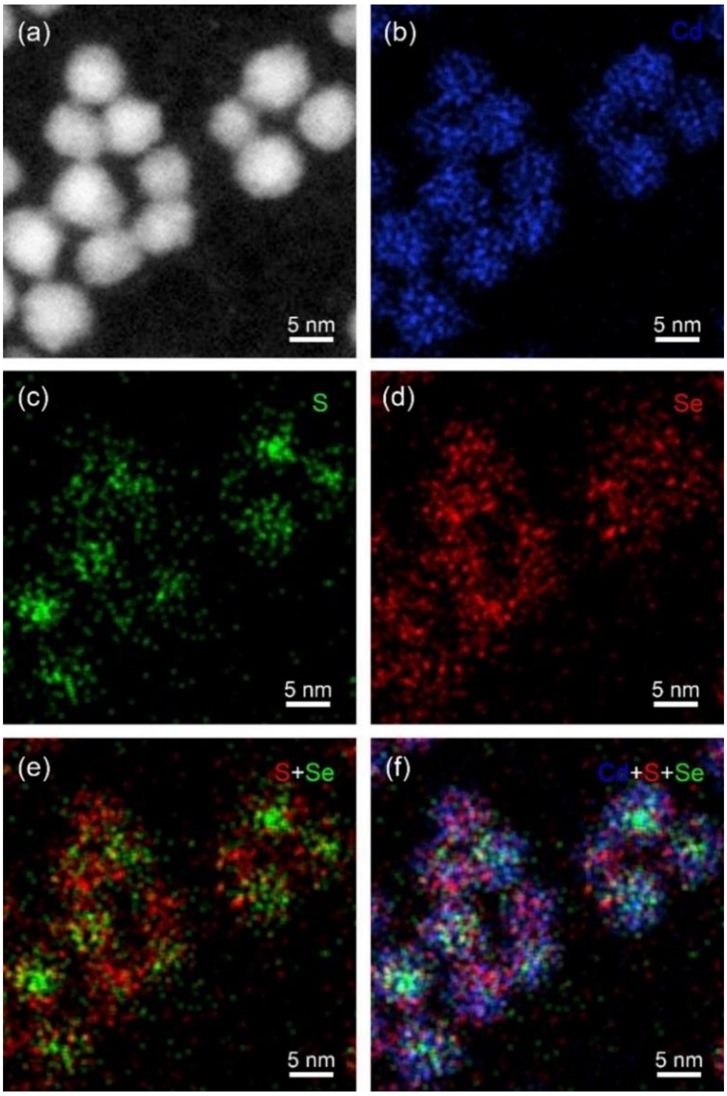

Figure S2. HAADF image (a) and EDS elemental maps (b-f) of CdSe@3CdS QDs.

Figure S3 shows the EDS elemental mapping of CdSe@9CdS QDs, which charts the atomic distributions of Cd, Se and S elements. Similar to Figure S2, the EDS elemental maps clearly show that Cd and S elements are distributed in the entire QDs, while Se is only present in the center of the nanocrystals. In addition, in Figure S3c, the contrast at the center region of the QDs is darker than that at the edges, indicating that the distribution of S elements in central region of the QDs is less than that in edge regions. From the overlapped mappings of Cd and S elements, and Cd, S and Se

elements (Figure S3e,f), one can clearly observe the distribution of elemental Se, intuitively confirming that the synthesized CdSe@CdS QDs are mainly core/shell heterostructure QDs.

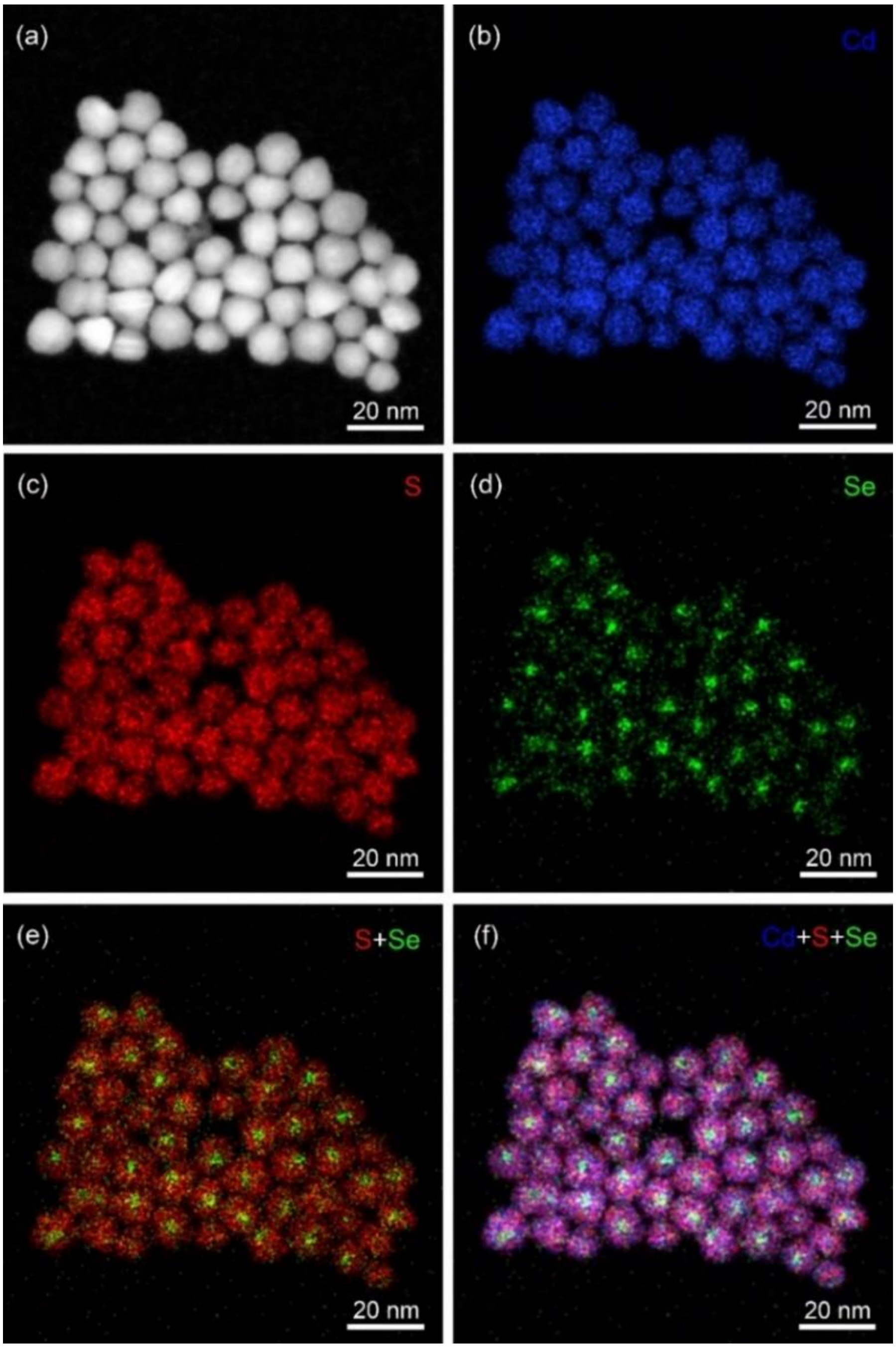

Figure S3. HAADF image (a) and EDS elemental maps (e-f) of CdSe@9CdS QDs.

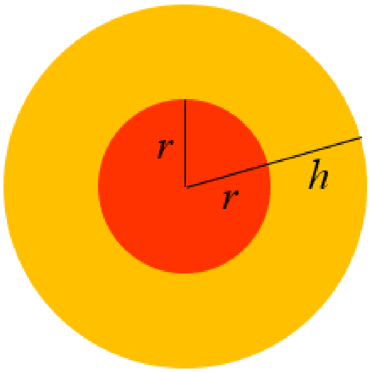

Figure S4. The projection of a core/shell spherical nanoparticle. The radius of the core is *r* and the shell thickness is *h*.

The circumference difference of shell and core is calculated as follows:

$$d = 2\pi(r+h) - 2\pi r = 2\pi h \quad (1)$$

When the circumference difference (d) of shell and core exceeds the misfit dislocation spacing (d_s), the ideal coherent interface will be destroyed. That means when $2\pi h \geq d_s$, the ideal coherent interface will be destroyed. The d_s can be calculated using the following formula[1]:

$$d_s = \frac{d_{CdSe} \times d_{CdS}}{d_{CdSe} - d_{CdS}} \quad (2)$$

where d_{CdSe} and d_{CdS} are the lattice spacing of CdSe and CdS on the same plane. Thus, the critical thickness of the shell (h_c) can be obtained using the following formula:

$$h_c = \frac{d_s}{2\pi} = \frac{d_{CdSe} \times d_{CdS}}{2\pi(d_{CdSe} - d_{CdS})} = \frac{d_{CdS}}{2\pi f} \quad (3)$$

where d_{CdS} is the lattice spacing of the CdS shell, and f is the lattice mismatch between the core and shell along a certain direction.

Reference:

[1] J. Zhang, W. Z. Wang, T. L. Wang, L. L. Jiang, N. Wang, Y. X. Dai, M. G. Wang, Y. Qi, *Ceram. Int.* **2021**, *47*, 8722.
